# Ambidextrous ungulates have more flexible behaviour, bolder personalities and migrate less

**DOI:** 10.1098/rsos.160958

**Published:** 2017-02-15

**Authors:** R. Found, C. C. St. Clair

**Affiliations:** Department of Biological Sciences, University of Alberta, Edmonton, Alberta, CanadaT6G 2R3

**Keywords:** laterality, habituation, adaptation, personality, migration, ungulates

## Abstract

Studies of wildlife have shown consistent individual variation in behavioural plasticity, which affects the rate of adaptation to changing environments. More flexible individuals may thus be more prone to habituation and conflict behaviour, but these applications of personality to wildlife management are little explored. Behavioural lateralization reflects cerebral specialization that may predict diverse expressions of behavioural plasticity. We recorded front-limb biases (i.e. handedness) in wild elk (*Cervus canadensis*), a species with facultative migration and high rates of habituation inside protected areas. Less lateralized elk responded more strongly to the application of aversive conditioning (predator-resembling chases by humans) by increasing their average flight response distances, but these same animals were also quicker to reduce their flight responses (i.e. habituate) when human approaches were benign. Greater laterality was correlated with, but not completely predicted by, bolder personalities, which we quantified via five correlated behavioural metrics. Lastly, lateralized elk were three times more likely to migrate, whereas less lateralized animals were similarly likely to remain near humans year-round. Lateralized behaviours can provide insight into behavioural flexibility enabling certain individuals to more quickly adapt to human-disturbed landscapes, and offer an especially productive arena for collaborative work by behaviourists, conservation biologists and wildlife managers.

## 1. Introduction

Wild populations typically respond to expanding human populations via one of avoidance, adaptation or exploitation (*sensu* [1]). Species that are more sensitive to human disturbance, such as grizzly bears (*Ursus arctos*) [2] and woodland caribou (*Rangifer tarandus*) [3] are especially likely to experience reductions in habitat availability and declining populations. Conversely, species that readily desensitize and habituate to human disturbance (as described in [4]), such as coyotes (*Canis latrans*) [5] and elk (*Cervus canadensis*) [6] can thrive, but in doing so often disrupt ecosystem function through local overpopulation and disrupted predator–prey dynamics [7]. This problem is particularly acute in protected areas where repetitive benign encounters with humans can accelerate the process of habituation [8], and prey species may exploit human-disturbed areas as predation refugia [9]. Habituated ungulates may also abandon migratory behaviour to use these human-disturbed areas year-round, thereby further damaging ecological integrity [10,11].

The rate at which a species tends to accommodate human-induced changes to the environment usually correlates negatively with the life-history trait of specialization (e.g. butterfly communities [12]), but similar variation can also exist among individuals within species (e.g. [13]). The persistence of behavioural or personality types within populations is presumed to result from environmental stochasticity, which selects for different suites of coevolved behavioural traits under different disturbance regimes [14]. If environmental change is rapid enough to occur within generations, selection may favour behavioural flexibility itself, for example, the individual variation in blue tit's (*Cyanistes caeruleus*) ability to adapt to changing predation and starvation risk [15]. Guilford [16] anticipated the adaptive benefit of this capacity for humans when he described creative and flexible individuals as ones that ‘respond efficiently and effectively to a constantly changing, and regularly challenging, environment’. This relative ability to adapt to, versus resist, change has been described more recently as coping style, and may complement personality to constitute a second axis of consistent individual behavioural variation (reviewed in [17]).

The behavioural flexibility that permits adaptation to changing environments is presumed to result from neural plasticity [18,19] and appears to be related by lateral differences in the brain. The compartmentalization of the brain into lateral hemispheres allows vertebrates—and even some invertebrates—to improve cognitive speed and efficiency [20,21]. Fitness benefits can accrue from both strong and weak laterality, resulting in the maintenance of individual variation in the degree of laterality [20]. Strong laterality (i.e. weak connections between hemispheres) has been correlated with quick responses to stimuli such as predators (e.g. by poeciliid fish, *Brachyraphis episcopi* [22]) or prey (e.g. by domestic dogs [23]), and in humans, increased physical coordination and autoimmune strength [24]. In contrast, weak laterality has been associated with superior learning ability [25] and creativity [26].

Measuring cerebral laterality in wild species can be challenging, but in a diversity of species, the strength of laterality can be inferred by observing lateralized behaviours, such as front-limb biases, which are largely independent of species-wide or even vertebrate-wide hemispherical specializations [27]. For example, if a ball is rolled straight towards an infant human, there is no obligate reason for the baby to grab it with one limb instead of the other. Weakly lateralized individuals are expected to choose to use their left and right limbs in similar proportions, while strongly lateralized individuals will consistently favour their left or right limb [28]. Front-limb biases have been reported in non-human species including felids, birds, amphibians and marsupials (reviewed in [29]).

Although it has not been investigated in this way, lateralization is likely to be relevant to many conservation problems stemming from rapid, human-caused changes to environments. These problems include the habituation of wildlife to people, which corresponds to the abandonment of migratory behaviour in some ungulate species. Although all ungulates exhibit some degree of flexibility in migratory behaviour [30], a gradual reduction in the proportion of animals that migrate appears to be occurring in ungulate species around the world, including wildebeest (*Connochaetes taurinus*) [31], Mongolian gazelles (*Procapra gutturosa*) [32], moose (*Alces alces*) [33] and elk [34]. Migratory behaviour has been associated with personality in a few diverse species, including a fish (*Rutilus rutilus*) [35], a bird (*Junco hyemalis*) [36] and, in our own previous work, a mammal (elk) [37]. In that study, we found that most migrating elk had shyer personality types, whereas most resident elk had bolder personality types. However, the presence of a few bold migrants and a few shy residents suggested that another factor pre-disposed individuals to their particular life-history choices. An opportunity to investigate the potential importance of laterality in the context is suggested by the presence of limb biases during grazing behaviour by feral horses [38] and a similar need for elk to paw craters in the snow to access forage in winter [39].

Here, we hypothesized that the tendency in elk to abandon migration and exhibit bold-type behaviour is associated with the weaker laterality of more behaviourally flexible individuals. An association between lateralization and migration might also explain why some elk continue to migrate, even though this can subject them to higher risk of predation [34]. Such life-history constraints were predicted to be a consequence of heritable variation in behavioural types [40], although there have been few demonstrations of this phenomenon. Our specific objectives were to (a) determine whether lateralization was evident in wild populations of elk comprised of both migratory and resident individuals, (b) identify potential correlations within individuals between laterality scores and quantifiable metrics of behavioural flexibility, and (c) compare the roles of laterality and personality in predicting the migratory strategies of elk. To achieve these objectives, we studied elk in two populations over two winters and quantified front-limb laterality when elk pawed at the snow, measured the responses of elk to repeated approaches by humans that were categorized as aversive or benign, and also compared their responses to a previously derived gradient of personality types.

## 2. Methods

### 2.1. Study areas and focal populations

All data were gathered in Banff and Jasper national parks, in the Canadian Rocky Mountains of Alberta, Canada, in the winters of 2010–2011 to 2012–2013. Banff is 6697 km^2^ in size, while northern neighbour Jasper is 10 880 km^2^ [41]. Each protected area has a human-disturbed townsite area exploited by elk for both anthropogenic forage and reduced predation from wolves (*Canis lupus*) and cougars (*Felis concolor*) [42]; J. Wilmshurst 2010, personal communication. We focused on large herds of adult females and sub-adults of both sexes that overwinter in the valley bottoms near the townsites within each park. Banff elk comprised a single group of 200–240 individuals each year, of which 36–50 adult females were marked with ear tags and very high frequency radio collars. Jasper elk divide into three neighbouring but non-mixing herds totalling 90–100 individuals, of which 22 adult females were marked with ear tags and/or radio collars. We identified ‘migrants’ as those individuals that migrated away from an identified winter range each spring, and ‘residents’ as those remaining within the winter range through to the next winter.

### 2.2. Lateralized behaviours

We recorded front-limb biases exhibited by elk when they accessed snow-covered grasses and forbs by digging and scraping through the snow with their front hooves. Elk can only use one hoof at a time for this behaviour, so we recorded whether they chose their left or right hoof for any single digging sequence, regardless of whether the sequence was a single strike, or the more usual occurrence of a few consecutive strikes. We only recorded front-limb biases by elk grazing on the level terrain because elk on the sloping terrain must use the downhill-facing limb for support. We calculated a value for laterality as the absolute strength of lateral biases, independent of side, using the equation |(L − R)/(L + R)|.

### 2.3. Behavioural flexibility

We used flight response distance as a metric of wariness that has previously demonstrated the capacity for behavioural change (reviewed in [43]; e.g. [44]). To measure flight response distance, we approached a targeted female elk at a steady walking pace from a minimum of 75 m away and only when elk were (a) at least 5 m from forest cover, (b) not bedded, and (c) not visibly engaged in any social interactions. We used a single observer for all flight response trials and recorded the distance at which elk responded to the approach by moving at least 5 m. While elk may have differences in original detection of our approaches, depending on which side we initially approached them from (e.g. horses [38]) and virtually all elk turned to face us before actually responding to our approach.

Our first metric of behavioural flexibility was derived from individual changes in wariness after elk were subjected to aversive conditioning (AC), from January through April 2012 (Jasper). Over several trials for each individual elk, we measured mean flight response distances before, during and after conditioning trials, and quantified the effect sizes of these changes in wariness. For detailed methodology and personality-based results of conditioning, see Found [45]. We performed AC on 18 elk, but because of natural mortality could only collect post-AC data on 17 elk. Eight months elapsed between the last of the AC trials and the first of the habituation trials (below), but we used the same individuals for each experiment.

Our second metric of behavioural flexibility was the degree to which individual elk habituate to repeated, benign approaches by humans. For each experimental trial, we recorded the change in elk flight response distance between two approaches separated by 7–10 min. The same observer performed each approach by walking towards the target elk from the same direction and with a similar pace and carriage, and returning to a blind after each approach. We approached elk from either their left or right side, or directly towards them, in roughly the same proportions, for each individual elk. The duration of time between the two approaches was chosen to provide an opportunity for elk to return to their previous behaviour, while retaining a high likelihood that an individual would remain in view and within the same social and environmental contexts for the second approach. We performed at least five sets of trials on each of 20 marked elk in Jasper, and 44 marked elk in Banff (2012 population), and recorded the mean change in flight responses between approaches as the ‘habituation response’ for each individual.

### 2.4. Personality

We compared the laterality gradient to a personality gradient derived previously [37] by using non-metric dimensional scaling (NMDS) to reduce a suite of separate, but correlated personality traits into a single dimension for each elk population and year (Banff; 2010, 2011 and 2012 and Jasper; 2012). This suite of separate behaviours included flight response distance, proportion of time spent vigilant, latency to respond to novel sound playbacks, central versus peripheral positions within the spatial herd structure, social position along a dominance hierarchy, leading behaviour and exploration of novel objects placed out in natural habitat.

### 2.5. Data analysis

We used Stata 11.1 (Statacorp) for all statistical analysis, and set *α* = 0.05. We used two-tailed *t*-tests to compare laterality means, and *χ*^2^-tests for all contingency and goodness-of-fit analyses of lateral biases. We used linear regression to compare the gradients of laterality to personality, and then applied logistic regression to determine whether migratory behaviour was better predicted by candidate models with the parameters for laterality, personality or a combination of the two. Because of the small number of parameters, we compared all possible models and ranked them using Akaike's information criterion (AIC). We assessed the fit of laterality and personality to migratory behaviour only for elk in the Banff population because it was a more complete mix of migrants and residents compared with Jasper, and also provided larger sample sizes. When reporting results of logistic regression we used Nagelkerke's pseudo-*R*^2^ values. We used generalized linear models (GLMs) to compare the effectiveness of laterality at predicting individual responses to benign versus aversive approaches by people and, for the Jasper elk that were more segregated according to migratory strategy, included migration as a random effect.

## 3. Results

### 3.1. Laterality

Elk in both populations exhibited front-limb biases, but the direction and magnitude of laterality differed among years and between populations. Including results for unmarked individuals, in Banff we recorded 6130 individual front-limb digging sequences in 2011 and 1292 sequences in 2012, and recorded 1469 sequences in Jasper (2012). In Banff there was a herd-wide bias for using the left front limb in 2011 (*χ*^2^-test: *χ*^2^ = 6.14, *p* = 0.013), and a contrasting but not statistically significant right front-limb bias in 2012 (*χ*^2^-test: *χ*^2^ = 3.51, *p* = 0.061). The herd-wide front-limb bias for elk in Jasper favoured the right foreleg (*χ*^2^-test: *χ*^2^ = 6.14, *p* = 0.013). The absolute magnitude of laterality for the marked elk differed among populations and years; it was greatest in Banff in 2012 (0.29 ± 0.047), similar in Jasper in 2012 (0.24 ± 0.036), but significantly lower in Banff in 2011 (0.15 ± 0.015; analysis of variance: *F*_2,106_ = 4.72, *p* = 0.011).

**Figure 1.**
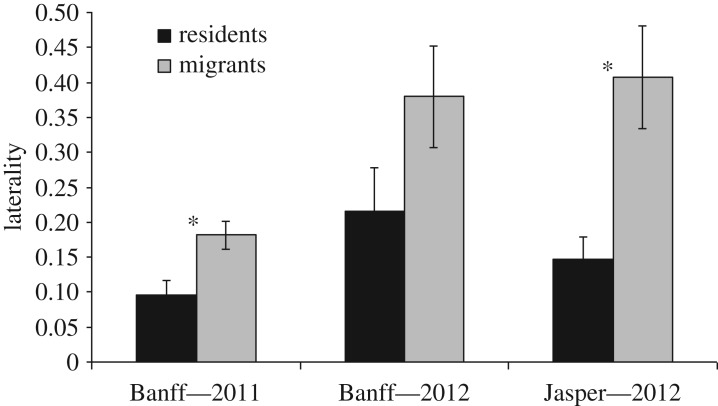
Comparison of behavioural lateralization in migratory (*n* = 29, 24, 7) and non-migratory (*n* = 21, 18, 13) elk in Banff (2011 and 2012) and Jasper (2012) National Parks, AB. Absolute strength of lateralized biases (*Y*-axis) calculated using |(L − R)/(L  +  R)|, from individual limb choices when elk dig through snow. Error bars represent s.e.

Migrants were more lateralized than residents were, but again with differences between the populations in direction and magnitude. In 2011 and Banff, the absolute values of laterality were almost 88% higher for migrants ($\bar{x}=0.18$) than residents ($\bar{x}=0.096$; *t*-test: *t*_1,48_ = 2.98, *p* < 0.005; figure 1). In 2012 and Banff, there was a similar result; migrants ($\bar{x}=0.38$) were 76% more lateralized than residents ($\bar{x}=0.22$; *t*-test: *t*_1,40_ = 1.66, *p* = 0.11). In Jasper, migrants ($\bar{x}=0.41$) were even more lateralized than residents (173%; 0.15; *t*-test: *t*_1,18_ = −3.85, *p* < 0.001). Migrants also expressed a greater directional limb bias than residents in Banff in 2011 (*χ*^2^-test: *χ*^2^ = 7.48, *p* = 0.042) and Jasper 2012 (*χ*^2^-test: *χ*^2^ = 4.63, *p* = 0.032). There was no difference in directional limb bias between migrants and residents in Banff in 2012 (*χ*^2^-test: *χ*^2^ = 0.16, *p* = 0.69).

### 3.2. Behavioural flexibility

**Figure 2.**
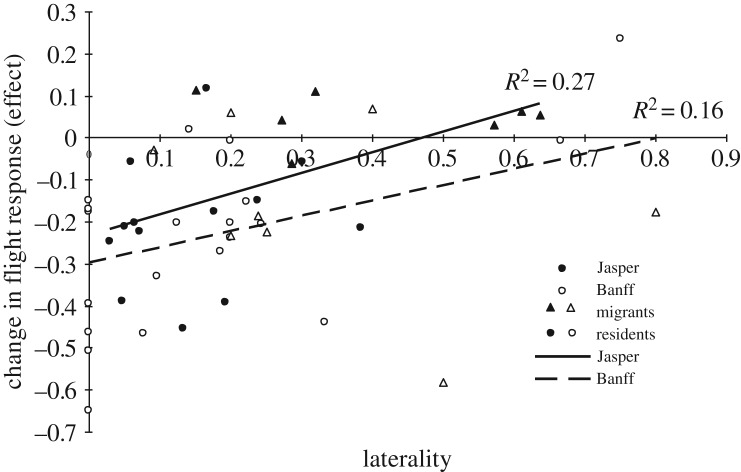
Correlation between laterality and mean elk responses to consecutive benign human approaches separated by 7–10 min, representing individual ‘habituation responses’. Laterality based on individual mean front-limb biases, using |(L − R)/(L  +  R)|. Trials were conducted on migratory/resident elk in Jasper (*n* = 7/13) and Banff (*n* = 14/15) National Parks, AB.

**Figure 3.**
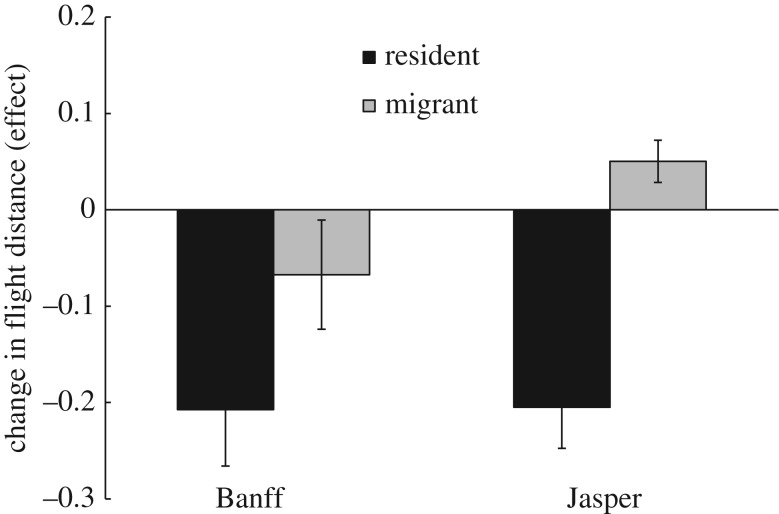
Mean effect sizes for elk changes in flight response distance between initial benign approach by human, and a second approach 7–10 min later. Data are from winter, 2012–2013, for migrants (*n* = 28, 7) and residents (*n* = 16, 13).

The absolute magnitude of laterality was significantly and negatively correlated with individual habituation responses to humans in both Jasper (GLM: *z*_18_ = 2.62, *p* = 0.009) and Banff (GLM: *z*_18_ = 2.19, *p* = 0.029; figure 2). To determine whether these results were confounded by the relationship between laterality and migratory strategy, we added migratory strategy as a random effect to the previous models, and found laterality was still correlated with habituation responses in Banff (*z*_18_ = 2.19, *p* = 0.029) but not in Jasper (GLM: *z*_18_ = 0.68, *p* = 0.50). In Jasper, the mean net change in flight response distance as a result of benign approaches by humans was negative in residents (−20.5 ± 4.2%), but positive and significantly smaller in magnitude in migrants (+5.0 ± 2.2%; *t*_1,18_ = −4.19, *p* < 0.001; figure 3). In Banff, habituation to benign approaches was similarly apparent in the decline in flight response distances for both residents (−20.7 ± 5.9%) and migrants (−6.7 ± 5.7%; *t*-test: *t*_1,42_ = 1.61, *p* = 0.12).

In addition to habituating more rapidly to benign approaches, less lateralized elk in Jasper also exhibited greater increases in wariness, via flight response distances, when exposed to aversive approaches by humans during predator-resembling chases (linear regression: *R*^2^ = 0.43, *F*_1,16_ = 11.19, *p* < 0.01). The mean change in flight distance was 62% greater in residents (+94.6 ± 11.3%) than migrants (+58.5 ± 14.6%; *t*-test: *t*_17_ = 1.40, *p* = 0.090). Habituation responses were best predicted by laterality models (Jasper and Banff), but responses to AC were best predicted by personality (Jasper; table 1).

**Table 1.** Generalized linear models for individual mean changes in flight response distance as a result of either ‘benign’ (in Banff and Jasper National Parks) or ‘aversive’ (Jasper only) stimuli.

treatment	model	ΔAIC	LL	*n*	*z*	*p*
benign^a^	laterality^c^	0	7.43	29	1.26	0.209
(Banff)	personality^d^	0.004	7.36	29	−1.20	0.230
benign	laterality	0	9.90	20	2.62	0.009
(Jasper)	personality	0.63	9.27	20	−2.31	0.021
aversive^b^	laterality	0.40	−2.92	17	−3.35	0.001
(Jasper)	personality	0	0.468	17	4.90	<0.001

^a^Consecutive passive human approaches separated by 7–10 min.

^b^10-min-long predator-resembling chases.

^c^Absolute front-limb biases for digging behaviour.

^d^Gradient of ‘boldness’ of personality type.

### 3.3. Laterality and personality as two axes of individual variation

We followed Found & St. Clair [37] to derive a personality gradient for the Banff 2012 elk population. This method uses NMDS to reduce the multiple personality traits comprising elk behavioural syndromes to two dimensions (Banff 2012; *n* = 53, loss criterion = 0.007). We used the first dimension to represent a gradient of personality types we labelled shy to bold, where bold personality types were socially and physically dominant, had shorter flight response distances, showed greater exploration of novel objects, adopted more peripheral positions within the herd, and exhibited slightly less vigilance behaviour. Personality gradients for Banff 2010 and 2011, and Jasper 2012, were from Found & St. Clair [37] directly.

**Figure 4.**
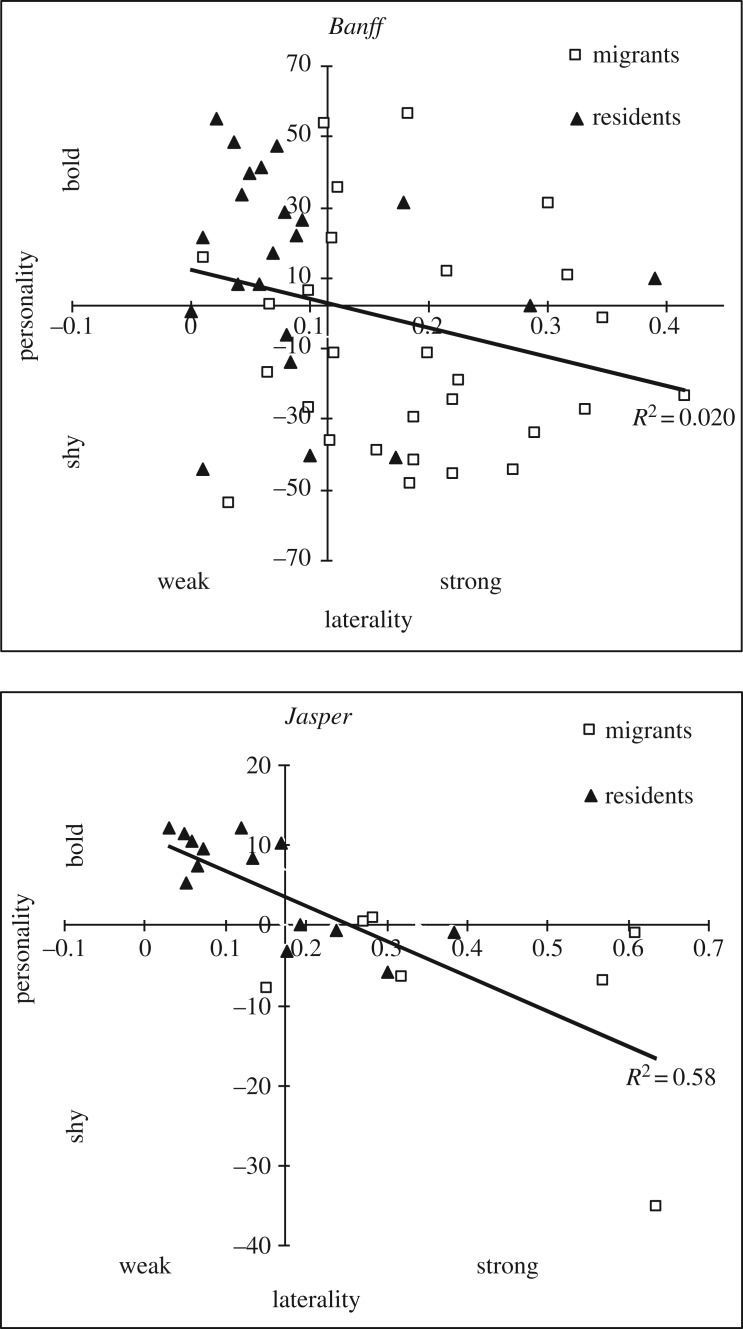
Correlations between gradients of laterality in wild elk in Banff (top) and Jasper (bottom) National Parks, AB. *X-* and *Y*-axes are at the global medians for each of laterality and personality, and define quadrants of weak versus strong laterality and shy versus bold personality type.

The association between laterality and personality differed for the two populations. There was a non-significant relationship between laterality and personality in Banff (linear regression: *R*^2^ = 0.020, *F*_49_ = 3.92, *p* = 0.054; figure 4), but a strong correlation between personality and laterality in Jasper (linear regression: *R*^2^ = 0.58, *F*_20_ = 26.43, *p* < 0.001; figure 4). Model comparisons showed that laterality was the better predictor of individual migratory strategies by elk in Banff, in both 2011 and 2012, but the personality model outranked the laterality model in Jasper (table 2).

**Table 2.** Logistic regression models for predicting migratory choices by elk in Banff and Jasper National Parks, AB using individual laterality or personality. Personality values are based on the delineation of behavioural syndromes into a single dimension. Laterality is the absolute value of the strengths of front-limb biases during winter digging behaviour.

model	study area	ΔAIC	*χ*^2^	L − L	*R*^2^^a^	*p*	res : mig^b^
personality	Banff 2010^c^	0	7.17	−18.54	0.162	<0.01	14 : 21
personality	Banff 2011	3.14	6.78	−31.11	0.098	<0.01	21 : 29
laterality	Banff 2011	0	9.93	−29.53	0.144	<0.005	21 : 29
personality	Banff 2012	0.12	3.25	−23.39	0.065	0.072	14 : 24
laterality	Banff 2012	0	3.36	−23.33	0.067	0.067	14 : 24
personality	Jasper 2012	0	11.13	−7.80	0.417	<0.001	14 : 7
laterality	Jasper 2012	0.09	11.04	−7.84	0.413	<0.001	14 : 7

^a^Nagelkerke's pseudo-*R*^2^.

^b^Number of individuals that were either resident or migrant.

^c^Laterality not recorded in 2010.

**Figure 5.**
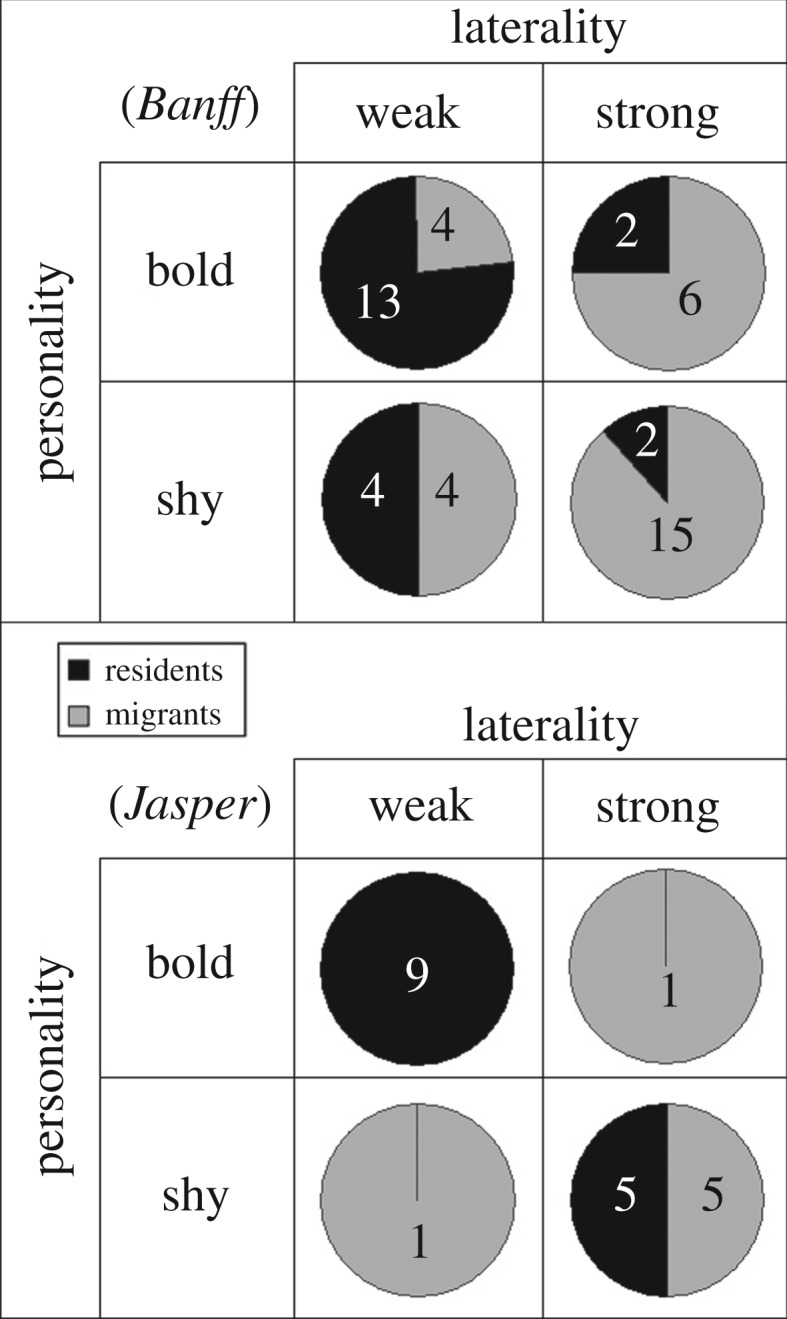
Numbers of migrant and resident elk in Banff (top) and Jasper (bottom) National Parks, classified by absolute magnitude of ‘laterality’, using front-limb biases and a ‘personality’ gradient derived from a behavioural syndrome with five separate personality traits.

We used the global medians for both laterality (Banff = 0.12, Jasper = 0.17) and personality (Banff = 2.0, Jasper = 0.17) to summarize variation among individuals in our populations in four quadrants describing laterality (weak or strong) and personality (shy or bold; figure 5). Migrants were found in the quadrant depicting weak and shy individuals more often that would be predicted by the independent assortment of these two variables in both Banff (93% higher than expected; *χ*^2^-test: $\chi_{3}^{2}=11.41$, *p* = 0.010) and Jasper (186% more than expected; *χ*^2^-test: $\chi_{3}^{2}=8.43$, *p* = 0.038). The complementarity of laterality and shyness resulted in occupancy of the complementary categories strong and shy or weak and bold for 68% of all elk in Banff and 90% in Jasper (figure 5).

## 4. Discussion

The migratory behaviour of ungulates around the world is gradually being replaced with year-round residency, often near human habitation. Our study explored the possibility that this change in behaviour is mediated by consistent individual variation in behaviour, as measured by gradients of behavioural flexibility and personality. We studied front-limb biases in foraging elk as proxies for individual variation in this flexibility and found that migratory elk in both Jasper and Banff were more strongly lateralized than resident elk. We used measures of habituation to benign approaches by humans, and sensitization to aversive chases, to reveal a predicted and negative correlation between behavioural flexibility and lateralization. In effect, the less lateralized individuals appeared to use the degree of threat evident in their earlier exposure to each kind of stimulus to moderate their subsequent response to that stimulus; demonstrating increased behavioural flexibility, but also perhaps increased learning ability. We found the strengths of correlations between gradients of personality type and laterality were weak in Banff, but moderate in Jasper, and that both metrics contributed to predictive models of migratory strategy.

Our study suggests that inherent differences in behavioural flexibility predict, in this population and potentially other ungulates, which individuals are more likely to habituate to people and cease migrating. We found that elk with greater flexibility in limb use were also more likely to exhibit year-round residency of townsites, which has been interpreted by others as an adaptation to human-disturbed areas [10,46]. Cerebral morphology may support this adaptation via flexibility that can apply to both limb use and migratory strategy. Some authors have suggested that adaptation to evolutionarily novel environments corresponds with a greater capacity to learn (e.g. [47]). This logically includes learning via habituation to ignore benign stimuli [48] thereby conserving the cost of wariness [49] and tailoring responsiveness to environmental context (*sensu* [50]). We reason that flexible individuals should habituate rapidly to people and their infrastructure in protected areas where human activities are likely varied, but generally benign [7]. In contrast, elk that migrate through predator-containing areas outside refugia (e.g. [34]) presumably benefit from stronger laterality partly because it preserves greater wariness to increase the likelihood of survival.

In addition to conserving wariness, individuals may benefit from greater lateralization because it speeds cerebral processing via one or both of sensitivity to particular stimuli [51] and the ability to multitask. Greater sensitivity to stimuli has been correlated with higher laterality in both dogs [23] and humans [52], to support the contrasting generalization that lesser sensitivity to stimuli results in more rapid habituation [48]. A second way for lateralization to speed cerebral processing could occur via a division of labour in the brain. Because the left cerebral hemisphere is specialized for processing routine foraging and social tasks, while the right is optimized for evaluating novel stimuli [53], more lateralized animals are better able to detect predators while foraging (e.g. chickens) [54]. This capacity to multitask may be particularly advantageous in ungulates [39]. Less lateralized animals that lack this ability may compensate with a greater tendency to actively assess risk in novel environments and adapt rapidly via habituation and other learned behaviours.

Laterality appears to delineate a gradient of individual variation in flexibility that partially overlaps with personality. Gradients of increasing boldness and decreasing lateralization correlated with responses to each of benign and aversive stimuli, but habituation responses were better explained by laterality, whereas responses to aversive stimuli were better explained by personality. One plausible reason for this difference is that the two traits are responsive at different temporal scales. In general, longer-term and more durable learning is associated with the development of personality [55] and previous aversive encounters may cause long-lasting behavioural change (e.g. [56]). Our AC trials were conducted over a longer period than our measurements of habituation responses, and so our methods may have unintentionally measured behavioural flexibility on two different time scales that are each best modelled with a different gradient of individual variation.

A second reason that personality better predicted responses to aversive over benign stimuli could be the overlapping domain of coping styles. These are typically characterized along a gradient of reactive–proactive behaviour in relation to stressors to define a unique, but overlapping, axis with metrics of personality (reviewed in [17]). In our study system, weakly lateralized elk had bolder personalities, which were expressed partly via greater neophilia in response to novel objects [37]. The complementarity of these tendencies—bold, neophilic and flexible—is expected of behavioural syndromes (*sensu* [14]) and potentially explains why most animals were either weakly lateralized and bold, or strongly lateralized and shy in each of Banff (68%) and Jasper (90%). Stronger associations in Jasper might be related to a higher density of predators there, more frequent interactions with humans or other factors that bear on the relative advantages of co-evolved behavioural tendencies. Similar associations between laterality and personality appear to have co-evolved in fish, including zebrafish (*Danio rerio*) [57] and rainbowfish (*Melanotaenia negrans*) [58]. These associations between behavioural types, laterality and predation risk may also be evident in fishes. Higher predation risk selected for strongly lateralized poeciliad individuals (*Brachyrhaphis episcopi*) [22], and also selected for shyer cyprinid individuals (*Rutilus rutilus*) [35].

Together, our results demonstrate high potential relevance of measuring both lateralization and personality in the context of wildlife management, especially for habituated animals that invoke human–wildlife conflict. We have shown that laterality can be measured with simple and non-invasive methods, even in wild animals. Such measures are already appreciated in the context of animal welfare as predictors of the dominant direction of fear responses, evidence of chronic environmental stress, and even individual temperament [59]. We suggest that this approach could make it possible to identify wild animals that are most prone to habituate while they are young enough to alter their behavioural trajectories, and before conflict intensifies. This approach could increase the efficacy of AC as a management technique, which is needed to meet the challenges of ever-expanding human populations that are increasingly intolerant of traditional methods such as lethal management [60].

Application of behavioural metrics to problems in wildlife management has the potential to advance a more basic understanding of variation in individual behaviour and to address many other pressing problems in conservation biology. There is a strong heritable component to each of personality [55] and laterality [61] and their coevolution is believed to constrain strategic life choices [62]. It follows that the rapidity with which human-dominated landscapes are changing exerts strong selective gradients on both the heritable and plastic components of these traits. It should be possible to predict the direction of the resulting selective gradients and the relative advantages of individuals with different sets of traits. For example, similar acknowledgements have already been applied to promoting greater success for captive breeding and reintroduction programmes [63], which supported the selection of bolder personalities for reintroduced black rhinos (*Diceros bicornis*) [64]. Similarly, both personality and lateralization could be relevant to anticipating which individuals will overcome the effects of climate change on forage availability (e.g. [65]) and predator–prey dynamics (e.g. [66]), or prevail in urban environments (following [36]). Most kinds of conservation threats favour bolder, more flexible individuals [67], which is likely to result in wildlife populations that are increasingly susceptible to habituation and conflict. Identifying those changes and anticipating their effects for both wildlife populations and people could make it possible for wildlife managers to target particular levels or combinations of boldness and lateralization in wildlife to promote their continued coexistence in human-dominated landscapes.

## Data Availability

The datasets supporting this article have been uploaded as https://dx.doi.org/10.6084/m9.figshare.4231820.v1.
